# Distinct tissue injury patterns in juvenile dermatomyositis auto-antibody subgroups

**DOI:** 10.1186/s40478-020-01007-3

**Published:** 2020-08-05

**Authors:** Mailan Nguyen, Vy Do, Paul C. Yell, Chanhee Jo, Jie Liu, Dennis K. Burns, Tracey Wright, Chunyu Cai

**Affiliations:** 1 Texas Children’s Hospital & Baylor College of Medicine, Houston, TX USA; 2 Department of Pediatrics, University of Texas Southwestern Medical Center, Dallas, TX USA; 3 Texas Scottish Rite Hospital for Children, Dallas, TX USA; 4 Department of Pathology, University of Texas Southwestern Medical Center, Dallas, TX USA

## Abstract

**Introduction:**

Juvenile dermatomyositis (JDM) can be classified into clinical serological subgroups by distinct myositis-specific antibodies (MSAs). It is incompletely understood whether different MSAs are associated with distinct pathological characteristics, clinical disease activities, or response to treatment.

**Methods:**

We retrospectively reviewed clinicopathological data from consecutive JDM patients followed in the pediatric rheumatology clinic at a single center between October 2016 and November 2018. Demographics, clinical data, and laboratory data were collected and analyzed. Detailed muscle biopsy evaluation of four domains (inflammation, myofiber, vessels, and connective tissue) was performed, followed by statistical analysis.

**Results:**

Of 43 subjects included in the study, 26 (60.5%) had a detectable MSA. The most common MSAs were anti-NXP-2 (13, 30.2%), anti-Mi-2 (7, 16.3%), and anti-MDA-5 (5, 11.6%). High titer anti-Mi-2 positively correlated with serum CK > 10,000 at initial visit (r = 0.96, *p* = 0.002). Muscle biopsied from subjects with high titer anti-Mi-2 had prominent perifascicular myofiber necrosis and perimysial connective tissue damage that resembled perifascicular necrotizing myopathy, but very little capillary C5b-9 deposition. Conversely, there was no positive correlation between the levels of the anti-NXP-2 titer and serum CK (r = − 0.21, *p* = 0.49). Muscle biopsies from patients with anti-NXP-2 showed prominent capillary C5b-9 deposition; but limited myofiber necrosis. Only one patient had anti-TIF1γ autoantibody, whose muscle pathology was similar as those with anti-NXP2. All patients with anti-MDA-5 had normal CK and near normal muscle histology.

**Conclusions:**

Muscle biopsy from JDM patients had MSA specific tissue injury patterns. These findings may help improve muscle biopsy diagnosis accuracy and inform personalized treatment of JDM.

## Introduction

Juvenile dermatomyositis (JDM) is the most common type of juvenile idiopathic inflammatory myopathy (IIM), and is characterized clinically by proximal muscle weakness, elevated muscle enzymes, and skin rashes in patients with onset before the age of 18. Classic skin rashes include Gottron papules and heliotrope rashes [1]. The calcinosis, gastrointestinal bleeding and ulcers, interstitial lung disease, or lipodystrophy seen in some patients highlights that juvenile DM is a systemic disease [2]. Diagnosis has been made based on the Bohan and Peter criteria for over four decades [3, 4], though new classification criteria have also been validated [5].

Recent advancements have shown that myositis-specific antibodies (MSAs) stratify patients into distinct phenotypes with implications regarding prognosis and treatment response [1]. Over half of patients with juvenile IIMs have at least one MSA [2, 6]. In patients with JDM, the anti-Mi2, anti-MDA-5, anti-NXP-2, and anti-TIF-1γ autoantibodies are the most common MSAs and are specific for dermatomyositis [2, 6]. Cases of myositis associated with anti-SRP and anti-HMGCR autoantibodies, in contrast, are classified as necrotizing autoimmune myopathy; and cases of myositis associated with Jo-1, PL-7 and PL-12 autoantibodies are classified as antisynthetase syndrome associated myositis [7, 8]. Patients in these latter two groups are no longer classified as dermatomyositis even if they have typical dermatomyositis type rashes per the most recent updates from the European Neuromuscular Center (ENMC) international workshop [7]. A unifying feature in all dermatomyositis subtypes is upregulation of type I interferon (IFN) signature genes, such as myxovirus resistant protein 1 (MxA) [9].

Muscle pathology may vary significantly depending on the type and titer of MSA. Yasin et al. reported histopathologic heterogeneity within the anti-Mi-2, anti-NXP-2, and anti-TIF-1γ groups and homogeneous minimal histopathologic change for the anti-MDA-5 group [10]. It would be beneficial to know the characteristics and range of pathology in the MSA subgroups, yet existing data on the MSA specific morphology are scarce. In this study, we performed detailed clinical, serological and pathological analyses in a cohort of consecutive JDM patients with muscle biopsies, and report MSA specific tissue injury patterns.

## Methods

### Study population

We retrospectively reviewed clinical charts of all patients aged 0–18 with a clinical diagnosis of definitive or probable juvenile DM according to the Bohan and Peter criteria [11], who were followed in the pediatric rheumatology clinic at a single center between October 2016 and November 2018. A diagnosis of JDM requires the presence of the characteristic skin changes of heliotrope rash and/or Gottron papules, and three (for definite JDM) or two (for probable JDM) of the following criteria: [1] symmetrical weakness of limb-girdle muscles and anterior neck flexors, progressing over weeks to months, with or without dysphagia or respiratory muscle involvement; (2) muscle-biopsy evidence of necrosis, phagocytosis, regeneration, perifascicular atrophy, inflammatory exudate, often perivascular; (3) elevation of skeletal-muscle enzymes in serum; (4) electromyography (EMG): short, small, polyphasic motor units, fibrillations, positive sharp waves, insertional irritability, high-frequency repetitive discharges.

Patients who did not have muscle biopsy samples available or had not been tested for MSAs were excluded. Altogether, 43 out a total of 130 patients fulfilled the inclusion/exclusion criteria and were included in the study.

Clinical data collection for markers of juvenile DM disease activity included muscle enzymes creatine kinase (CK), aldolase, lactate dehydrogenase, aspartate aminotransferase, and alanine transaminase, magnetic resonance imaging (MRI) findings. Clinical weakness was assessed by the Childhood Myositis Assessment Scale (CMAS) [12] and the Manual Muscle Testing in 8 muscles (MMT-8) [13]. CMAS is an observational performance-based assessment of 14 functional tasks to assess muscle endurance, muscle function, strength, with a total score range from 0 to 52. MMT-8 evaluated neck flexors, deltoids, biceps, wrist extensors, gluteus maximus and medius, quadriceps and ankle dorsiflexors, each muscle scored from 0 to 10, with total score range 0–80. Treatment regimens received by patients at 6 months were also collected.

### Myositis-specific antibody screening

The MSA tests were performed by Quest Diagnostics, a CLIA approved commercial laboratory. The Quest Diagnostics Myositis Specific 11 Antibodies Panel is a line blot assay that screens for anti-Mi-2, anti-MDA-5, anti-TIF-1γ, anti-NXP-2, anti-Jo-1, anti-PL-7, anti-PL-12, anti-EJ, anti-OJ, and anti-SRP. For each antibody, a result of > 11 SI is considered abnormal/positive. The test has been validated for clinical use. More information is available at the company test website: https://testdirectory.questdiagnostics.com/test/test-detail/94777/myositis-specific-11-antibodies-panel?cc=MASTER

### Muscle histologic analyses

In new patients suspected of having JDM, most had their muscle biopsy taken within 1–2 weeks of initial evaluation, concurrent with MSA testing. They were not under chronic immunosuppression, though some might have received 1 or 2 rounds of pulse-dose steroids. Referred or recurrent patients typically had muscle biopsy taken months to years before MSA testing. Muscle biopsies performed prior to 2016 were scored but excluded from statistical analyses, as those biopsies were taken > 1 year before MSA testing and may not accurately represent muscle pathology at time of MSA testing.

Routine enzyme histochemical stains were performed on slides/micrographs from the CLIA approved neuropathology clinical laboratory at UT Southwestern Medical Center, included H&E, Gomori trichrome, nicotinamide adenine dinucleotide-tetrazolium reductase (NADH-TR), myosin ATPases performed at pH 9.4, pH 4.6 and pH 4.3, acid phosphatase, alkaline phosphatase, esterase, succinate dehydrogenase (SDH), cytochrome C oxidase (COX). Immunostaining included MHC Class I (US Biological, M3886–10, Salem, MA), C5b-9 (Dako/Agilent, terminal complement complex, clone aE11, Santa Clara, CA), and CD3 (Dako/Agilent, clone F7.2.38).

All slides were reviewed by two neuropathologists, including a practicing neuromuscular pathology specialist (CC) and a neuropathology trainee (PY). Initial muscle pathology assessment was performed independently by the two raters, following the recommendations from the International Consensus Group on Juvenile DM [14], and yielded suboptimal interrater agreement (< 70% agreement). Slides with discrepant results were reviewed and discussed at a multi-head scope. The scoring criteria were subsequently modified and specified in Table 1. The final scores for all parameter were reached by consensus agreement.

**Table 1:** Histological score tool for muscle biopsy pathology. TRI: tubuloreticular inclusions. EM: electron microscopy. MHC: major histocompatibility complex

Domain	Score	Definitions and Instructions
**INFLAMMATORY DOMAIN**		
CD3+ endomysial infiltration	0,1,2	For each of endomysial, perimysial, perivascular distributions, score for CD3+ infiltrating cells as follows: if none, or < 4 cells in a × 20 field = score 0; if≥4 cells in a 20x field and/or1 cluster (cluster is 10 cells or more) = score of 1; if ≥2 clusters in whole biopsy and/or diffuse infiltrating cells (i.e. > 20 cells in a 20x field) = score of 2.
CD3+ perimysial infiltration	0,1,2	
CD3+ perivasclar infiltration	0,1,2	
Macrophage endomysial infiltration	0,1,2	For each of endomysial, perimysial, perivascular distributions, score for macrophage, by acid phosphatase stain: if none, or < 4 cells in a ×20 field = score 0; if ≥4 cells in a 20x field and/or1 cluster (cluster is 10 cells or more) = score of 1; if ≥2 clusters in whole biopsy and/or diffuse infiltrating cells (i.e. > 20 cells in a 20x field) = score of 2.
Macrophage perimysial infiltration	0,1,2	
Macrophage perivascular infiltration	0,1,2	
**VASCULAR DOMAIN**		
Capillary endothelial TRI by EM	0,1,2	Score = 0 if no TRI; score = 1 if < 2 TRI/10 examined capillaries; score = 2 if ≥2TRI/10 examined capillaries.
C5b-9 positive capillaries	0,1,2	scores = 0 if none, score = 1 if rare, score = 2 if multifocal and robust
Arterial abnormality	0,1	Mural thickening and/or endothelial swelling and/or transmural inflammation in arteries/arterioles. Absence = 0, presence = 1.
Infarction	0,1	Well demarcated regional loss of myofiber nuclei and loss of normal cytoarchitecture. Absence = 0, presence = 1.
**MUSCLE FIBER DOMAIN**		
MHC Class I over expression	0,1	Presence of MHC1 in myofibers. Absence = 0, presence = 1.
Atrophy perifascicular	0,1,2	Affecting > 6 myofibers out of 10 along one edge of a fascicle, not exclusively type II fibers. 0 = absent, 1 = present in one or two fascicles, 2 = present in 3 or more fascicles.
Atrophy nonperifascicular	0,1	Atrophy of nonperifascicular myofibers: 0 = absent, 1 = present.
C5b-9 positive necrotic fiber	0,1,2	Myofibers with strong sarcoplasmic C5b-9 expression: 0 = absent, 1 if < 6 positive fibers in a 20x field, 2 if ≥6 positive fibers in a 20x field.
Degenerating fibers perifascicular	0,1,2	Includes focal basophilia within a fiber, vacuolation, sarcoplasmic rarification, and/or pallor, myophagocytosis, acid phosphatase positive fibers, alkaline phosphatase positive fibers): 0 = absent, 1 = present in one or two fascicles, 2 = present in three or more fascicles
Degenerating fibers non-perifascicular	0,1,2	
Internal nuclei	0,1	Internal nuclei in non-basophilic otherwise normal fibers: 0 if < 3% of fibers, 1 if > 3% of fibers.
**CONNECTIVE TISSUE DOMAIN**		
Endomysial fibrosis	0,1	Assessed on Masson’s trichrome stain: 0 = absent, 1 = present
Perimysium alkaline phosphatase reactivity	0,1,2	Alkaline phosphatase connective tissue reactivity, excluding arteries/arterioles: 0 = absent, 1 = present, but weak and focal, 2 = strong and widespread.

### Electron microscopy

Electron microscopy (EM) examination for endothelial tubuloreticular inclusions were available for 39 or 43 cases. For each case, thick sections from four resin blocks were evaluated by light microscopy. One block was selected for thin section grid. For EM, the entire specimen area was systematically scanned at lower power on EM. Capillaries, preferentially those with swollen endothelium, were examined at higher magnification for TRI. Typically at least 10 capillaries were examined at high magnification. In cases with no or rare TRI, at least 20 capillaries were examined at high magnification.

### Statistical analysis

Statistical analyses were focused on the patient groups with the three most frequent MSA subtypes: anti-Mi-2, anti-MDA-5, and anti-NXP-2. Nonparametric tests including the Mann-Whitney test (for two groups) or the Kruskal-Wallis test (for three groups) were used to compare histological features in MSA subgroups. The Pearson correlation coefficient (r) was calculated to analyze the degree of correlation between the titers of MSA subtypes and CK. For these examinations *p* < 0.05 was considered statistically significant.

## Results

### Demographic and serologic features of the cohort

Demographic and serologic features of the patients are summarized in Table 2. Among the 43 patients included in this study, 74% were female. The racial distribution was 41.9% Hispanic, 39.5% white, 14% black, and 4.6% of other races. All patients by inclusion criteria had characteristic DM skin changes of heliotrope rash and/or Gottron papules. Only 1 patient with positive MDA5 autoantibody had interstitial lung disease. Sixty percent (*n* = 26) had a single detectable MSA. The most prevalent MSAs were anti-NXP-2 (*n* = 9, 20.9%), anti-Mi-2 (*n* = 5, 11.6%), and anti-MDA-5 (*n* = 3, 7.0%). Anti-SRP, anti-Tif-1γ, and anti-PL-7 were detected but uncommon (Table 2). 14% of patients had multiple detectable MSAs. Of the three most prevalent single MSA types, patients with anti-Mi-2 had the lowest MMT-8 scores at presentation (Table 2), indicating most severe weakness. Patients with anti-MDA5 had the highest, near normal MMT-8 scores. Patients with anti-NXP-2 had intermediate, variable MMT-8 scores. The difference was statistically significant (*p* = 0.0417 by Kruskal-Wallis test). Mi-2 patient also showed a trend of presenting with the lowest average CMAS scores, although this was more variable and not reaching statistical significance (*p* = 0.1151).

**Table 2:** Demographics, serologic types and clinical weakness in patients included in the analyses (*n* = 43)

*Demographics and MSA types*		*CMAS* *Mean (range)*	*MMT8* *Mean (range)*	*CK (U/L)* *Mean (range)*
Age at diagnosis, years	6 (2–16)			
**Sex**	*n (%)*			
Male	11 (25.6)			
Female	32 (74.4)			
**Race**	*n (%)*			
Hispanic	18 (41.9)			
White	17 (39.5)			
Black	6 (14.0)			
Other	2 (4.6)			
**MSA**	*n (%)*			
Anti-NXP-2	9 (20.9)	21 (0–51)	59 (32–80)	1019 (47–4681)
Anti-Mi-2	5 (11.6)	15 (0–46)	25 (21–30)	10,366 (1491-18,992)
Anti-MDA-5	3 (7.0)	49 (46–52)	72 (64–80)	37 (13–50)
Anti-Tif-1γ	1 (2.3)	32	59	402
Anti-SRP	1 (2.3)	48	76	132
Anti-PL-7	1 (2.3)	2	45	13,972
Multiple MSAs	6 (14.0)	40 (32–48)	30 (21–39)	1022 (93–3860)
No MSA	17 (39.5)	27 (4–46)	55 (36–76)	2548 (26–12,809)

### Serum Mi-2 titer directly correlates with CK level

**Fig. 1:**
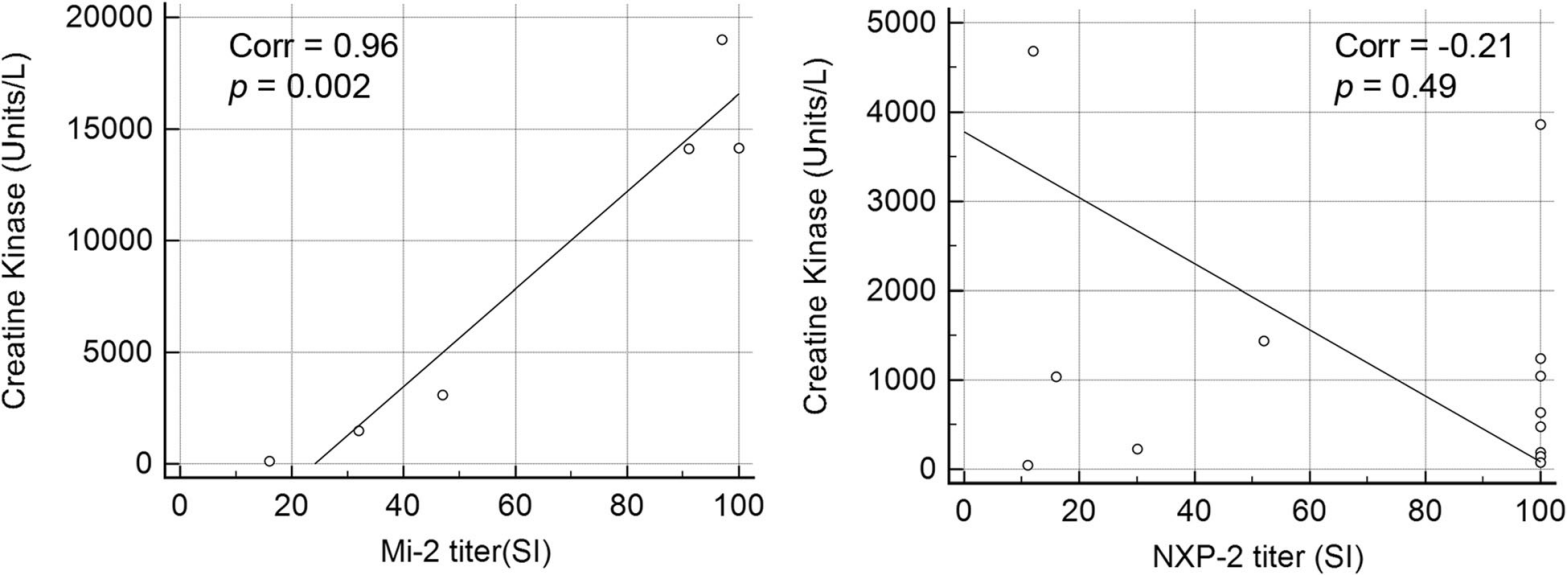
Correlation dot plots illustrated a strong positive correlation between Mi-2 titer and serum CK (*r* = 0.96, *p* = 0.002), but no correlation between NXP-2 titer and serum CK (*r* = −0.21, *p* = 0.49)

The average initial CK level in all cases was 2913 (26–18,992) U/L. In patients with only one MSA, the average CK level was 10,366 (1491-18,992) U/L for the Mi-2 cohort, 1019 (47–4681) U/L for the NXP-2 cohort, and 37 (13–50) U/L for the MDA-5 cohort. In the Mi-2 cohort, there was a direct correlation between the titer of the anti-Mi-2 autoantibody and the serum CK level at initial visit (r = 0.96, *p* = 0.002) (Table 3; Fig. 1a). Patients with greater than 90 KU/L anti-Mi-2 titer all had greater than 10,000 U/L serum CK, the highest in our cohort. With the exception of one patient with anti-PL-7, no other MSA subtypes had a CK greater than 5000 at the initial visit. A lower anti-Mi-2 titer was associated with lower CK levels. Using the initial MMT-8 score, the patients with anti-Mi-2 exhibited more muscle weakness compared to the NXP-2 group (*p* = 0.03). In comparison, in the NXP-2cohort, there was no correlation between anti-NXP-2 titer and CK (r = − 0.21, *p* = 0.49) (Table 3. Figure 1b). High anti-NXP-2 titers were seen in patients with either normal or elevated CK. Similarly, low anti-NXP-2 titer were seen in patients with either normal or elevated CK. Patients with positive anti-MDA-5 all had normal CK. Only one patient had high anti-TIF-1γ antibody, and he had mildly elevated CK.

**Table 3:** MSA type, titer, and CK level in patients with positive MSA (n = 26)

	AGE	SEX	MSA TYPE	Mi-2 titer	NXP-2 titer	MDA-5 titer	TIF1γ titer	SRP titer	CK
1	10	M	MDA5			50			126
2	5	M	MDA5			50			103
3	12	F	MDA5			13			88
4	12	F	MI2	≥100					14,152
5	4	F	MI2	97					18,992
6	16	F	MI2	91					14,119
7	4	F	MI2	47					3077
8	5	F	MI2	32					1491
9	2	F	NXP2		≥100				475
10	3	M	NXP2		≥100				74
11	12	F	NXP2		≥100				1044
12	13	F	NXP2		≥100				147
13	8	F	NXP2		52				1438
14	3	F	NXP2		30				229
15	4	M	NXP2		16				1040
16	8	F	NXP2		12				4681
17	10	F	NXP2		11				47
18	12	F	TIF1γ				84		402
19	12	F	PL7						13,972
20	8	F	SRP					24	132
21	4	M	SRP, TIF1γ				13	58	93
22	4	F	NXP2, MDA5		≥100	34			3860
23	5	F	NXP2, MI2	98	≥100				633
24	3	F	NXP2, OJ		≥100				1242
25	3	M	NXP2, SRP		≥100			12	190
26	3	F	MI2, MDA5	16		11			117

### Characteristic histopathologic findings based on myositis-specific antibody group

In new patients suspected of having JDM, most had their muscle biopsy taken within 1–2 weeks of initial evaluation, concurrent with MSA testing. They were not under chronic immunosuppression, though some might have received 1 or 2 rounds of pulse-dose steroids. Referred or recurrent patients typically had muscle biopsy taken months to years before MSA testing. Pathology of those cases were scored but not included into statistical analysis as they may not accurately represent muscle pathology at the time of MSA testing.

**Fig. 2:**
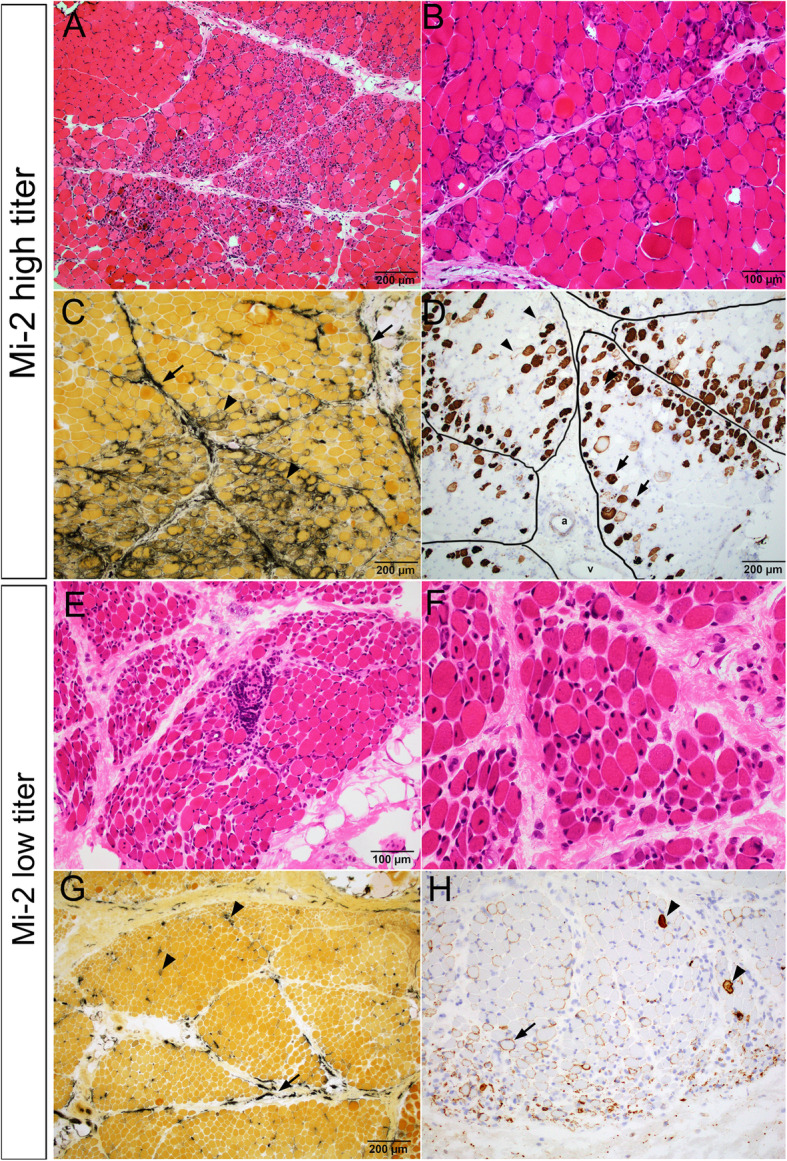
Characteristic muscle pathology in patients with high and low titers of Mi-2 auto-antibody. **a**-**d** Patient 4 with high Mi-2 titer. **a** Low and **(b)** high power images of H&E stained cryosections showed prominent perifascicular myofiber necrosis and regeneration. **c** Alkaline phosphatase stain highlighted frequent regenerating fibers (arrow heads) as well as strong perimysial connective tissue reactivity (arrows). **d** C5b-9 immunostain showed frequent necrotic fibers concentrated in the perifascicular regions (arrow heads) but might present at the center of fascicles. Viable but injured myofibers might show sarcolemmal C5b-9 reactivity (arrow heads). There was no significant capillary C5b-9 reactivity. Perimysium was outlined by black lines. “**a**” indicate perimysial artery. “**v**” indicate perimysial vein. **e**-**h** Patient 8 with low Mi-2 titer. **e** Low and (**f**) high power H&E showed prominent perifascicular atrophy and frequent internal nuclei, but few acutely necrotic fibers. **g** Alkaline phosphatase stain highlighted rare regenerating fibers (arrow heads) and patchy discontinuous perimysial connective tissue reactivity (arrows). **h** C5b-9 immunostain highlighted occasional necrotic fibers (arrow heads) but rather wide spread sarcolemmal C5b-9 reactivity in viable myofibers (arrow heads). There was no significant capillary C5b-9 reactivity

In patients with one MSA antibody, three patients with high Mi-2 titers all had prominent perifascicular myofiber necrosis (Fig. 2a-b). Acutely necrotic fibers were pale on H&E and often intermixed with basophilic regenerating fibers. The perimysial connective tissue showed strong and widespread alkaline phosphatase reactivity, often extending well beyond regions with active myofiber necrosis. The alkaline phosphatase stain also highlighted regenerating fibers in these regions (Fig. 2c). The acutely necrotic fibers show strong sarcolemmal reactivity for C5b-9 (Fig. 2d). The C5b-9 positive fibers were most concentrated at the perifasicular region, and decreasing towards the center of fascicles. Many viable perifascicular fibers also showed sarcolemmal C5b-9 reactivity, similarly with a gradient of being more prominent at the perifascicluar regions and decreasing towards the center. Quantitatively, 61 ± 8.8% of the C5b-9 sarcoplasm positive necrotic fibers were located in the perifascicular region, defined as the two penultimate layers of myofibers immediately adjacent to perimysium [8]. This perifascicular necrotizing myopathy pattern was highly reminiscent of muscle pathology in Jo-1 myositis [8, 15], although myofiber necrosis in Mi-2 positive patients were less restricted to the perifascicualr regions and could extend well into the center of the fascicules (Fig. 2a, d). Capillary C5b-9 deposition was minimal to absent, although capillary endothelial cell tubuloreticular inclusions were easy to find in most Mi-2 cases.

There was a positive correlation between the serum CK level and the number of necrotic fibers in the Mi-2 cohort. Patients with low anti-Mi-2 titers and low CK had many fewer necrotic myofibers (Fig. 2h), although a significant subset of viable myofibers showed sarcolemmal C5b-9 reactivity. Alkaline phosphatase reactivity was also much reduced and present in a more patchy distribution. (Fig. 2g).

**Fig. 3:**
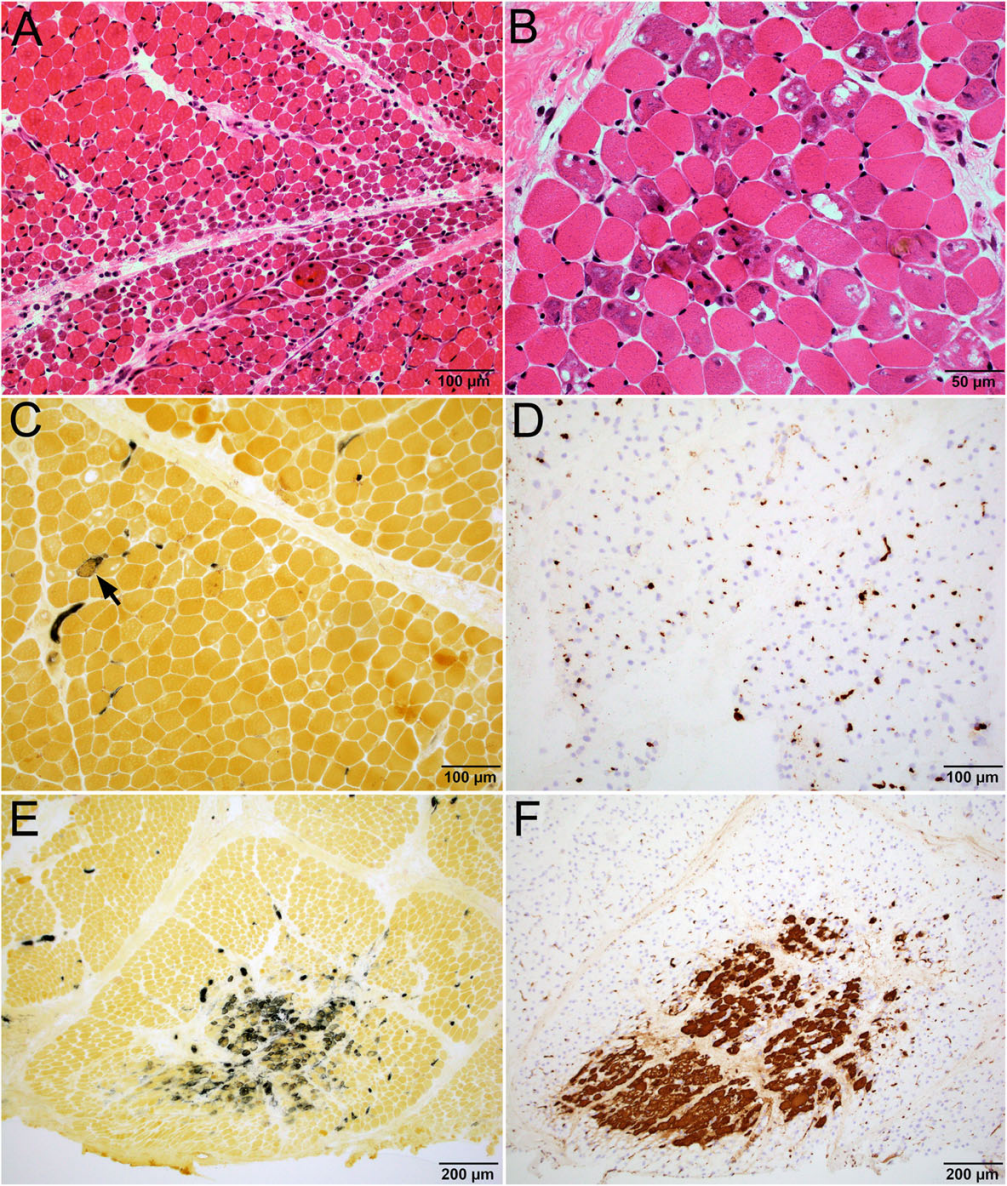
Characteristic muscle pathology in patients with high titers of NXP-2 auto-antibody. **a**-**f** Patient 9 with high NXP-2 titer. **a** Low power image of H&E stained cryosections showed perifascicular atrophy. High power image (**b**) showed that many myofibers had basophilic vacuolar degeneration. **c** Alkaline phosphatase stain highlighted only rare regenerating fiber; the basophilic vacuolar fibers were non-reactive. There was no abnormal connective tissue reactivity. **d** C5b-9 immunostain highlighted prominent capillary reactivity, but no necrotic fibers or sarcolemmal C5b-9 reactivity. Panels (**e**) alkaline phosphatase and (**f**) C5b-9 showed a focus of infarction with grouped necrotic and regenerating fibers at the center of a fascicle

In patients with anti-NXP-2 autoantibody, there was no correlation between anti-NXP2 titer and the extent of myofiber damage. Myofiber damages were variable and when present, manifested as sarcoplasmic vacuolation and basophilia rather than frank necrosis (Fig. 3a, b). These fibers were not reactive for either the regenerating fiber marker alkaline phosphatase (Fig. 3c) or the necrotic fiber marker C5b-9 (Fig. 3d). Connective tissue alkaline phosphatase activity was absent or focal (Fig. 3c). The five patients with high and moderate anti-NXP-2 titers had prominent C5b-9 deposition in capillaries. The capillary C5b-9 deposition were patchy, most robust in the perifascicular regions and decreased towards the center of the fascicles. (Fig. 3d). One (patient 9) had an ischemic infarct at the center of a fascicle (Fig. 3e, f), which was likely secondary to vascular injury rather than direct immune attack on muscle fibers. In the four patients with moderate to low anti-NXP-2 titer, muscle biopsies were all taken prior to 2016, thus might not reflect muscle pathology at the time of MSA testing. Of those, two cases with moderate anti-NXP-2 titers (Table 3, patients 13,14) showed basophilic vacuolar degeneration of myofibers and patchy strong capillary C5b-9 deposition. The two cases with low/border-line positive anti-NXP-2 titer showed perifascicular atrophy only without active myofiber damage (case 15) and normal histology (case 16), respectively. Overall, the NXP-2 cohort cases showed same pattern of injury with different levels of severity that corresponded to the serum anti-NXP-2 titers.

All patients with anti-MDA-5 had normal or near normal muscle histology, with findings limited to focal perimysial MHC1 upregulation and mildly increased interstitial macrophages in one case. One patient had anti-TIF1γ autoantibody, whose pathology was similar to NXP-2 patients, with prominent capillary C5b-9 deposition, basophilic and vacuolated myofibers, but no overt myofiber necrosis. Of the 6 patients with mixed autoantibodies, 3 (patients 22, 24, 25) had high titer NXP-2 combined with one other autoantibody at borderline titers. The muscle pathology of these cases were generally consistent with NXP2 pathology. Patient 26 had borderline Mi2 and MDA5 autoantibodies, her muscle biopsy was normal. In those cases the borderline values should probably be considered negative. One outlier was patient 23, with high titers for both NXP2 and Mi-2. Her muscle unexpectedly showed minimal capillary C5b-9 deposition and no C5b-9 positive necrotic fibers, raising possibility of an erroneous result.

The single patient with anti-PL-7 showed abundant C5b-9 positive necrotic fibers that were scattered throughout but accentuated in the perifascicular region, with no significant capillary C5b-9 reactivity. Alkaline phosphatase showed widespread perimysial connective tissue reactivity. EM showed frequent well-formed endothelial tubuloreticular inclusions. These features were similar to Mi-2 group muscle pathology, and also consistent with the “necrotizing perifascicular myositis” reported in patients with Jo-1 autoantibody [8]. The sole patient with low titer SRP antibody showed rare, randomly scattered necrotic fibers, and no capillary C5b-9 reactivity or endothelial tubuloreticular inclusions.

In the 17 patients with no detectable MSA, 8 patients had normal or near normal muscle morphology; 5 had perifascicular atrophy without significant acute myopathic changes, 2 had perifascicular necrotizing myopathy, and the remaining 2 cases had randomly distributed necrotic and regenerating fibers. The findings suggest that a large subset of the no MSA group might represent patients in remission with low/undetectable antibody levels, while small groups of active patients with yet unidentified autoantibody and/or other types of myositis might also be present.

### Statistical analysis of histological features

Twenty one histological features were scored and compared among the patients with anti-NXP-2, anti-Mi-2 or anti-MDA-5 mono-autoantibodies and concurrent muscle biopsies (Table 4). Kruskal-Wallis test among all three MSA groups showed statistically significant differences in the extent of endomysial macrophages, perifascicular myofiber degeneration, capillary C5b-9, myofiber C5b-9, and perimysial connective tissue alkaline phosphatase reactivity. When comparing between the NXP-2 and Mi-2 groups using Mann-Whitney test, the NXP-2 group was more likely to have C5b-9 positive capillaries (*p* = 0.0277), while the Mi-2 group was more likely to have C5b-9 positive myofibers (*p* = 0.0308) and perimysial connective tissue alkaline phosphatase reactivity (*p* = 0.0284). There was a trend for the Mi-2 group to have more prominent endomysial macrophage inflammation (*p* = 0.0632).

**Table 4:** Pathology features in muscle biopsies from JDM patients with Mi2, NXP2 and MDA5 autoantibodies

Histology	Mi2 (*n* = 4)	NXP2 (*n* = 4)	MDA5 (*n* = 2)	*p* value K-W^1^	*p* value M-W^2^
CD3 endomysial (0/1/2)	3/1/0	2/2/0	2/0/0	0.4720	0.5357
CD3 perimysial (0/1/2)	1/0/3	1/0/3	2/0/0	0.1850	1.0000
CD3 perivascular (0/1/2)	0/1/3	1/0/3	2/0/0	0.1083	0.8687
Macrophages endomysial (0/1/2)	0/0/4	0/3/1	1/1/0	**0.0377**	0.0632
Macrophages perimysial (0/1/2)	0/1/3	0/3/1	1/1/0	0.1136	0.2248
Macrophages perivascular (0/1/2)	1/2/1	0/3/1	2/0/0	0.1228	0.6446
Capillary TRI (EM) (0/1/2)	0/1/2	0/0/4	2/0/0	0.0970	0.3778
Arterial abnormal (0/1)	0/4	1/3	2/0	0.0554	0.4497
Infarction (0/1)	4/0	3/1	2/0	0.4724	0.4497
MHC-1 upregulation (0/1)	0/4	0/3	1/1	0.1738	1.0000
Atrophy perifascicular (0/1/2)	0/1/3	0/1/3	2/0/0	0.0601	1.0000
Atrophy non-perifascicular (0/1)	2/2	3/1	2/0	0.4724	0.5357
Type IIc fibers (0/1)	1/3	1/3	2/0	0.1850	1.0000
Degenerating^a^ fibers perifascicular (0/1/2)	0/1/3	0/3/1	2/0/0	**0.0498**	0.2248
Degenerating^a^ fibers non-perifascicular (0/1/2	2/1/1	1/2/1	2/0/0	0.3221	0.6612
Internal nuclei (0/1)	2/2	3/1	2/0	0.4724	0.5357
Fibrosis endomysial (0/1)	2/2	3/1	2/0	0.4724	0.5357
Fibrosis perimysial (0/1)	1/3	1/3	2/0	0.1850	1.0000
ALK perimysium (0/1/2)	0/1/3	3/1/0	2/0	**0.0271**	**0.0284**
C5b-9 myofiber (0/1/2)	0/0/3	1/3/0	2/0/0	**0.0219**	**0.0308**
C5b-9 capillary (0/1/2)	2/1/0	0/0/4	2/0/0	**0.0180**	**0.0277**

^1^: *p*-values calculated by Kruskal-Wallis test among the Mi-2, NXP-2 and MDA5 mono-autoantibody groups. Patients with multiple autoantibodies were excluded

^2^: *p*-values calculated by Mann-Whitney test between the Mi-2 and NXP-2 mono-autoantibody groups. Patients with multiple autoantibodies were excluded

^a^: These include degenerating fibers, regenerating fibers and necrotic fibers

## Discussions

The recent advances in the recognition of MSAs have re-defined dermatomyositis [7]. However, clinical information on MSA status is often not available to pathologists evaluating muscle biopsies, and very little is known about MSA specific muscle pathology. In this article, we report distinct tissue injury patterns in the most common MSA types in patients with JDM, which may help improve muscle biopsy diagnosis accuracy and inform patient care. Mi-2 was associated with acute myofiber necrosis and connective tissue damage in a predominantly perifascicular distribution. These characteristic features were prominent when the anit-Mi-2 titer was high and much less pronounced when the anti-Mi-2 titer was low. There was a direct positive correlation between the anti-Mi-2 titer, serum CK and the extent of myofiber necrosis. On the other hand, patients with NXP-2 autoantibody had prominent capillary C5b-9 deposition. Myofiber damages were often limited to vacuolar-basophilic degeneration rather than frank necrosis; there was no direct correlation between NXP-2 titer and serum CK. Patients with anti-TIF-1γ had very similar muscle pathology as those with NXP-2. Patients with anti-MDA-5 in our cohort all had normal serum CK at time of diagnosis and normal muscle histology, consistent with previous reports of amyopathic or hypomyopathic disease [2, 6].

We found alkaline phosphatase enzyme histochemical stain and C5b-9 immunostain particularly helpful in differentiating the pathology of JDM MSA groups. Alkaline phosphatase stain relies on endogenous alkaline phosphatase activity to hydrolyze exogenous alpha-naphthyl acid phosphate substrate to form a black reaction product in the presence of fast blue RR salt [16]. In skeletal muscle, alkaline phosphatase reactivity is normally only present in the endothelium of arterioles, but not in capillaries, myofibers or connective tissue [17]. The main use of alkaline phosphatase in skeletal muscle biopsy is to highlight regenerating myofibers [18], connective tissue injury [8, 19] and abnormal capillaries [20]. In this study, we found that muscles in the anti-Mi-2 positive group had more widespread and stronger alkaline phosphate reactivity than the anti-NXP-2 positive group, indicating more perimysial connective tissue damage. Pestronk described this injury pattern as immune myopathies with perimysial pathology (IMPP) [21], which can be seen in Jo-1 myositis [8, 21], other antisynthetase syndrome associated myositis with autoantibodies such as PL-12, EJ, necrotizing autoimmune myopathy with HMGCR auto antibody [22], and patients with SSA/SSA52 autoantibody [19]. Remarkably, 98% of those patients with IMPP muscle pathology had a sustained beneficial response to immunomodulatory therapies [19]. Likewise, a study on 101 patients from the UK Juvenile Dermatomyositis Cohort reported that JDM patients with Mi-2 autoantibody were 7 fold less likely to remain on treatment over time, despite having more severe muscle pathology on initial biopsy [23]. Taken together, these data suggest that anti-Mi-2 patients characteristically demonstrate an IMPP muscle injury pattern on alkaline phosphatase stain, which may predict a more sustained response to immunomodulatory therapies.

C5b-9 is an immunohistochemical stain that labels the terminal complement complex/membrane attacking complex. In normal muscle, C5b-9 is only seen in the wall of perimysial arteries, which serves as a useful internal positive control. In myopathic conditions, three pathological C5b-9 staining patterns have been described: sarcoplasmic, sarcolemmal, and capillary. Strong sarcoplasmic C5b-9 expression labels any acutely necrotic fibers irrespective of etiology [24]. Sarcolemmal C5b-9 expression is a feature of damaged but viable myofibers, and has been reported in a wide range of myopathic conditions including Jo-1 myositis [8], some muscular dystrophies [25], X-linked vacuolated myopathy [26], and necrotizing autoimmune myopathy [27]. Capillary C5b-9 expression is a characteristic finding in dermatomyositis [24]. Although it is not completely specific and have also been reported in muscles from diabetic patients with poor glycemic control [28] and myopathy with anti-SRP autoantibody [29]. The distribution of C5b-9 positive capillaries is more concentrated in the perifascicular region in dermatomyositis, but more diffuse/random in the latter two conditions. In our cohort of JDM patients, all patients with anit-NXP-2 or anti-TIF1γ autoantibodies had prominent capillary C5b-9 deposition, while patients with anti-Mi-2 and anti-MDA5 autoantibodies had minimal to no capillary C5b-9 deposition. There is only one patient in our JDM cohort with TIF1γ antibody, which may not be sufficient data on this category to draw generalized conclusion. Hida etal have also reported 36 adult patients with positive TIF1 antibody, whose characteristic muscle pathology was “vacuolated fibers and dense Cb-9 deposits on capillaries” [30], very similar to the TIF1γ positive patients in our JDM cohort.

It has been suggested that the pathogenesis of dermatomyositis involves a complement-mediated microangiopathy with destruction of capillaries, which in turn leads to hypoperfusion of the myofibers, particularly those in the perifascicular regions, resulting in perifascicular myocyte necrosis, phagocytosis, and atrophy [24, 31]. Several lines of evidence, however, challenge this hypothesis. First, widespread C5b-9 deposition in capillaries is a very common finding in the muscle and nerve of patients with poorly controlled diabetes [28, 32]. However, with the exception of localized diabetic muscle infarction, myofiber necrosis in these patients is rare and they nearly never develop perifascicular atrophy or other morphological features of inflammatory myopathy. Conversely, patients with anti-Jo-1 myositis [8] and systemic sclerosis with anti-PM-Scl auto-antibody [33] also characteristically demonstrate perifascicular myofiber necrosis and atrophy, but no significant C5b-9 deposition or capillary dropout have been reported in these patients. Second, the extent of capillary C5b-9 expression did not correlate with the amount of myofiber necrosis in our JDM cohort. Finally, it is difficult to attribute perifascicular atrophy to hypoperfusion of the feeding artery/arteriole, in that arterial territories are typically round and often cross the boundary of perimysium. Obstruction of these feeding vessels leads to regional infarctions at the center of a fascicle, as seen in Fig. 3f, and in patients with paraneoplastic necrotizing myopathy [34], rather than selective injury of perifascicular myofibers. Taken together, these evidence suggest that microangiopathy may not be the sole mechanism that lead to myofiber damage and atrophy in dermatomyositis. Rather, our finding of MSA specific tissue injury patterns leads us to speculate that there might be tissue specific receptors for the different types of MSA. Mi-2 autoantibody may preferentially bind to myofibers, while NXP-2 and TIF1γ autoantibodies may preferentially bind to capillaries. Activation of these various receptors all result in upregulation of the interferon (IFN) pathway genes [35], and downstream complement pathway activation.

This study is limited as a relatively small, retrospective, single institution study. The absence of long term follow-up data precludes the possibility of comparing long term treatment responses in different MSA subgroups. Longitudinal studies on a larger cohort is desirable to confirm whether perimysial alkaline phosphatase reactivity predicts better prognosis in patients with anti-Mi-2 autoantibody. Statistical analyses were performed on cases with concurrent MSA testing and muscle biopsy only, further limiting sample size, particularly the MDA5 group. The majority of the pathological features demonstrated a trend to occur less in the MDA5 group, which may prove statistically significant in larger cohort studies.

## Conclusions

To our knowledge, this is the first paper to report a positive correlation between the anit-Mi-2 titer, serum CK level, and muscle pathology in JDM patients. It is also the first to describe a perifascicular necrotizing myopathy/IMPP pattern in patients with anti-Mi-2 autoantibody that is morphologically similar to Jo-1 myositis. Awareness of this morphological resemblance may prove useful to pathologists to avoid misclassifying these cases into Jo-1 myositis. Identification of MSA specific tissue injury patterns may lead to more personalized, MSA specific treatment of JDM in the future.

## Data Availability

The datasets during and/or analyzed during the current study is available from the corresponding author on reasonable request.

## References

[1] Rider LG, Katz JD, Jones OY (2013) Developments in the classification and treatment of the juvenile idiopathic inflammatory myopathies. Rheum Dis Clin North Am 39(4):877–904 10.1016/j.rdc.2013.06.001 PMC3817412 24182859

[2] Pachman LM, Khojah AM (2018). Advances in juvenile dermatomyositis: myositis specific antibodies aid in understanding disease heterogeneity. J Pediatr.

[3] Bohan A, Peter JB (1975). Polymyositis and dermatomyositis (second of two parts). N Engl J Med.

[4] Bohan A, Peter JB (1975). Polymyositis and dermatomyositis (first of two parts). N Engl J Med.

[5] Lundberg IE, Tjarnlund A, Bottai M, Werth VP, Pilkington C, Visser M (2017). 2017 European league against rheumatism/American College of Rheumatology classification criteria for adult and juvenile idiopathic inflammatory myopathies and their major subgroups. Ann Rheum Dis.

[6] Rider LG, Nistala K (2016) The juvenile idiopathic inflammatory myopathies: pathogenesis, clinical and autoantibody phenotypes, and outcomes. J Intern Med 280(1):24–38 10.1111/joim.12444 PMC4914449 27028907

[7] Mammen AL, Allenbach Y, Stenzel W, Benveniste O (2019) 239th ENMC international workshop: classification of dermatomyositis. In: Neuromuscular disorders : NMD, Amsterdam, 14-16 December Neuromuscular Disorders 30(1):70-92 10.1016/j.nmd.2019.10.005 31791867

[8] Mescam-Mancini L, Allenbach Y, Hervier B, Devilliers H, Mariampillay K, Dubourg O (2015). Anti-Jo-1 antibody-positive patients show a characteristic necrotizing perifascicular myositis. Brain.

[9] Uruha A, Nishikawa A, Tsuburaya RS, Hamanaka K, Kuwana M, Watanabe Y (2017). Sarcoplasmic MxA expression: a valuable marker of dermatomyositis. Neurology..

[10] Yasin SA, Schutz PW, Deakin CT, Sag E, Varsani H, Simou S et al (2019) Histological heterogeneity in a large clinical cohort of juvenile idiopathic inflammatory myopathy: analysis by myositis autoantibody and pathological features. Neuropathol Appl Neurobiol 45(5):495-512 10.1111/nan.12528 PMC6767402 30378704

[11] Hoogendijk JE, Amato AA, Lecky BR, Choy EH, Lundberg IE, Rose MR (2004). 119th ENMC international workshop: trial design in adult idiopathic inflammatory myopathies, with the exception of inclusion body myositis, 10-12 October 2003, Naarden, the Netherlands. Neuromuscular Disorders.

[12] Lovell DJ, Lindsley CB, Rennebohm RM, Ballinger SH, Bowyer SL, Giannini EH (1999). Development of validated disease activity and damage indices for the juvenile idiopathic inflammatory myopathies. II. The childhood myositis assessment scale (CMAS): a quantitative tool for the evaluation of muscle function. The juvenile dermatomyositis disease activity collaborative study group. Arthritis Rheum.

[13] Rider LG, Koziol D, Giannini EH, Jain MS, Smith MR, Whitney-Mahoney K (2010). Validation of manual muscle testing and a subset of eight muscles for adult and juvenile idiopathic inflammatory myopathies. Arthritis Care Res.

[14] Wedderburn LR, Varsani H, Li CK, Newton KR, Amato AA, Banwell B (2007). International consensus on a proposed score system for muscle biopsy evaluation in patients with juvenile dermatomyositis: a tool for potential use in clinical trials. Arthritis Rheum.

[15] Mozaffar T, Pestronk A (2000). Myopathy with anti-Jo-1 antibodies: pathology in perimysium and neighbouring muscle fibres. J Neurol Neurosurg Psychiatry.

[16] Burns DK (2020) Skeletal muscle biopsy evaluation. In: Zhou L, Burns D, Cai C (eds) A case-based guide to neuromuscular pathology. Cham Springer, 10.1007/978-3-030-25682-1_1

[17] Romanul FC, Bannister RGJN (1962) Localized areas of high alkaline phosphatase activity in endothelium of arteries. Nature 195(4841):611-2 10.1038/195611a0 14493577

[18] Engel WK, Cunningham GGJJH (1970). Alkaline phosphatase-positive abnormal muscle fibers of humans. Cytochemistry.

[19] Bucelli RC, Pestronk A (2018). Immune myopathies with perimysial pathology: clinical and laboratory features. Neurol(R) Neuroimmunol Neuroinflamm.

[20] Cros D, Pearson C, Verity MA (1980). Polymyositis-dermatomyositis: diagnostic and prognostic significance of muscle alkaline phosphatase. Am J Pathol.

[21] Pestronk A (2011). Acquired immune and inflammatory myopathies: pathologic classification. Curr Opin Rheumatol.

[22] Alshehri A, Choksi R, Bucelli R, Pestronk A (2015). Myopathy with anti-HMGCR antibodies: perimysium and myofiber pathology. Neurol(R) Neuroimmunol Neuroinflamm.

[23] Deakin CT, Yasin SA, Simou S, Arnold KA, Tansley SL, Betteridge ZE (2016). Muscle biopsy findings in combination with myositis-specific autoantibodies aid prediction of outcomes in juvenile dermatomyositis. Arthritis Rheumatol (Hoboken, NJ).

[24] Kissel JT, Mendell JR, Rammohan KW (1986). Microvascular deposition of complement membrane attack complex in dermatomyositis. N Engl J Med.

[25] Spuler S, Engel AG (1998). Unexpected sarcolemmal complement membrane attack complex deposits on nonnecrotic muscle fibers in muscular dystrophies. Neurology..

[26] Louboutin JP, Navenot JM, Villanova M, Rouger K, Merlini L, Fardeau M (1998). X-linked vacuolated myopathy: membrane attack complex deposition on the surface membrane of injured muscle fibers is not accompanied by S-protein. Muscle Nerve.

[27] Christopher-Stine L, Casciola-Rosen LA, Hong G, Chung T, Corse AM, Mammen AL (2010). A novel autoantibody recognizing 200-kd and 100-kd proteins is associated with an immune-mediated necrotizing myopathy. Arthritis Rheum.

[28] Yell PC, Burns DK, Dittmar EG, White CL, Cai C (2018). Diffuse microvascular C5b-9 deposition is a common feature in muscle and nerve biopsies from diabetic patients. Acta Neuropathol Commun.

[29] Miller T, Al-Lozi MT, Lopate G, Pestronk A (2002). Myopathy with antibodies to the signal recognition particle: clinical and pathological features. J Neurol Neurosurg Psychiatry.

[30] Hida A, Yamashita T, Hosono Y, Inoue M, Kaida K, Kadoya M (2016). Anti-TIF1-γ antibody and cancer-associated myositis: a clinicohistopathologic study. Neurology..

[31] Dalakas MC (2015). Inflammatory muscle diseases. N Engl J Med.

[32] Rosoklija GB, Dwork AJ, Younger DS, Karlikaya G, Latov N, Hays AP (2000). Local activation of the complement system in endoneurial microvessels of diabetic neuropathy. Acta Neuropathol.

[33] De Lorenzo R, Pinal-Fernandez I, Huang W, Albayda J, Tiniakou E, Johnson C (2018). Muscular and extramuscular clinical features of patients with anti-PM/Scl autoantibodies. Neurology..

[34] Cai C, Alshehri A, Choksi R, Pestronk A (2014). Regional ischemic immune myopathy: a paraneoplastic dermatomyopathy. J Neuropathol Exp Neurol.

[35] Pinal-Fernandez I, Casal-Dominguez M, Derfoul A, Pak K, Plotz P, Miller FW (2019). Identification of distinctive interferon gene signatures in different types of myositis. Neurology..

